# Supplementary figures and images for: Pencil graphite as electrode platform for free chlorine sensors and energy storage devices
Source: PLoS One. 2021 Mar 11;16(3):e0248142. doi: 10.1371/journal.pone.0248142 (PMC7951880; doi:10.1371/journal.pone.0248142)

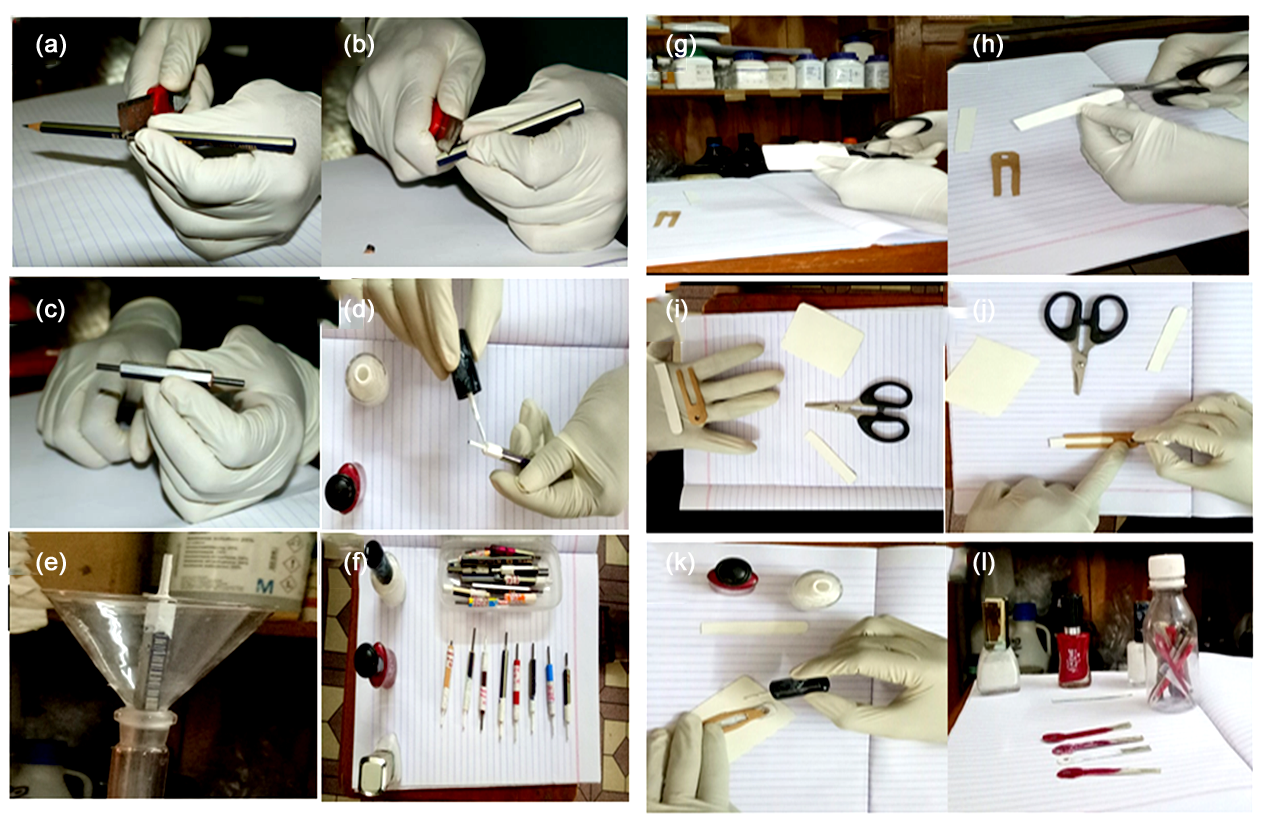

Supplement: S1 Fig — Stepwise fabrication of PGE (a-f), and PDPE (g-l). (TIF) [file pone.0248142.s001.tif]

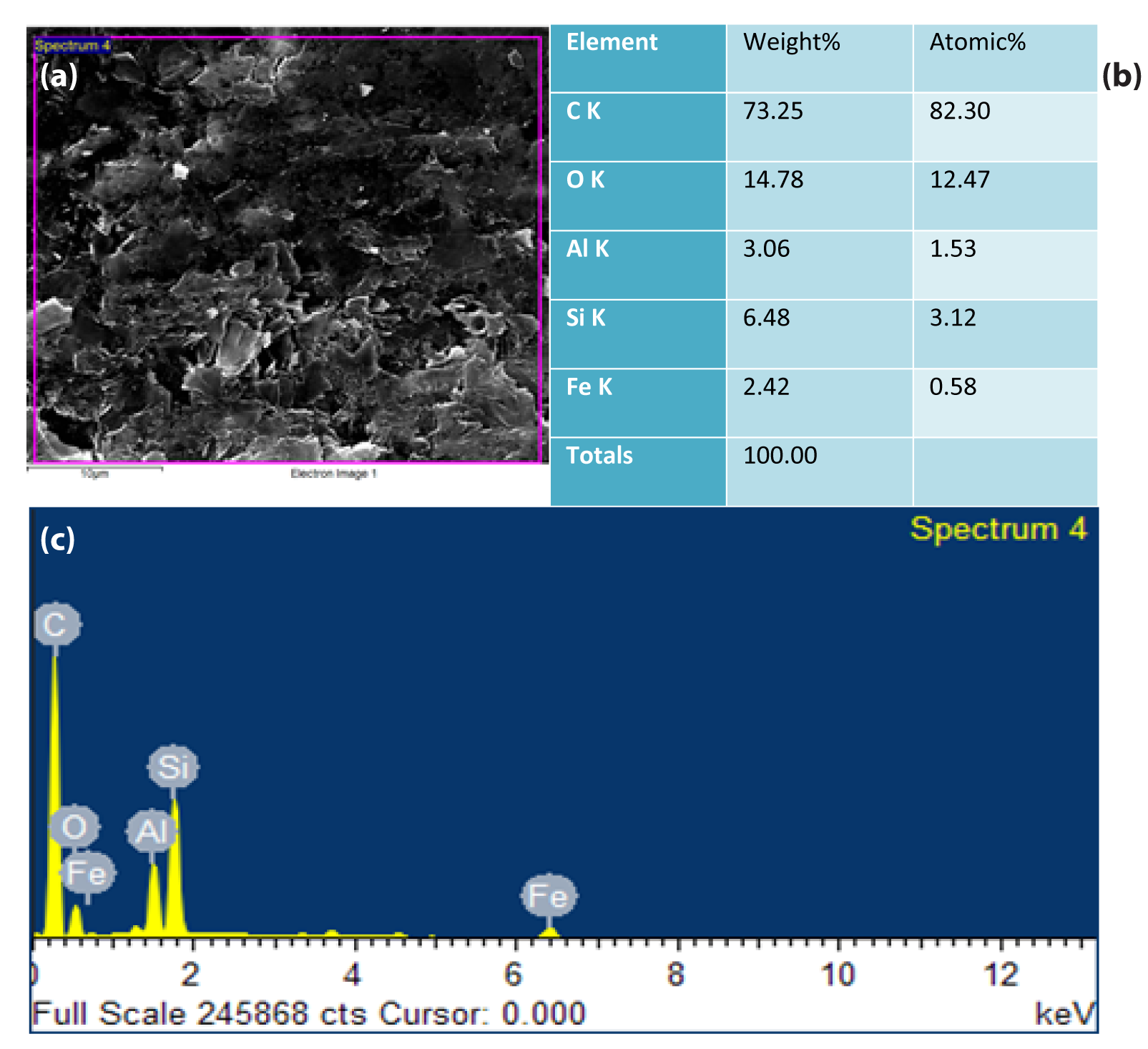

Supplement: S2 Fig — (a) SEM of PGE; (b) Composition on the PGE surface; (c) EDX of PGE. (TIF) [file pone.0248142.s002.tif]

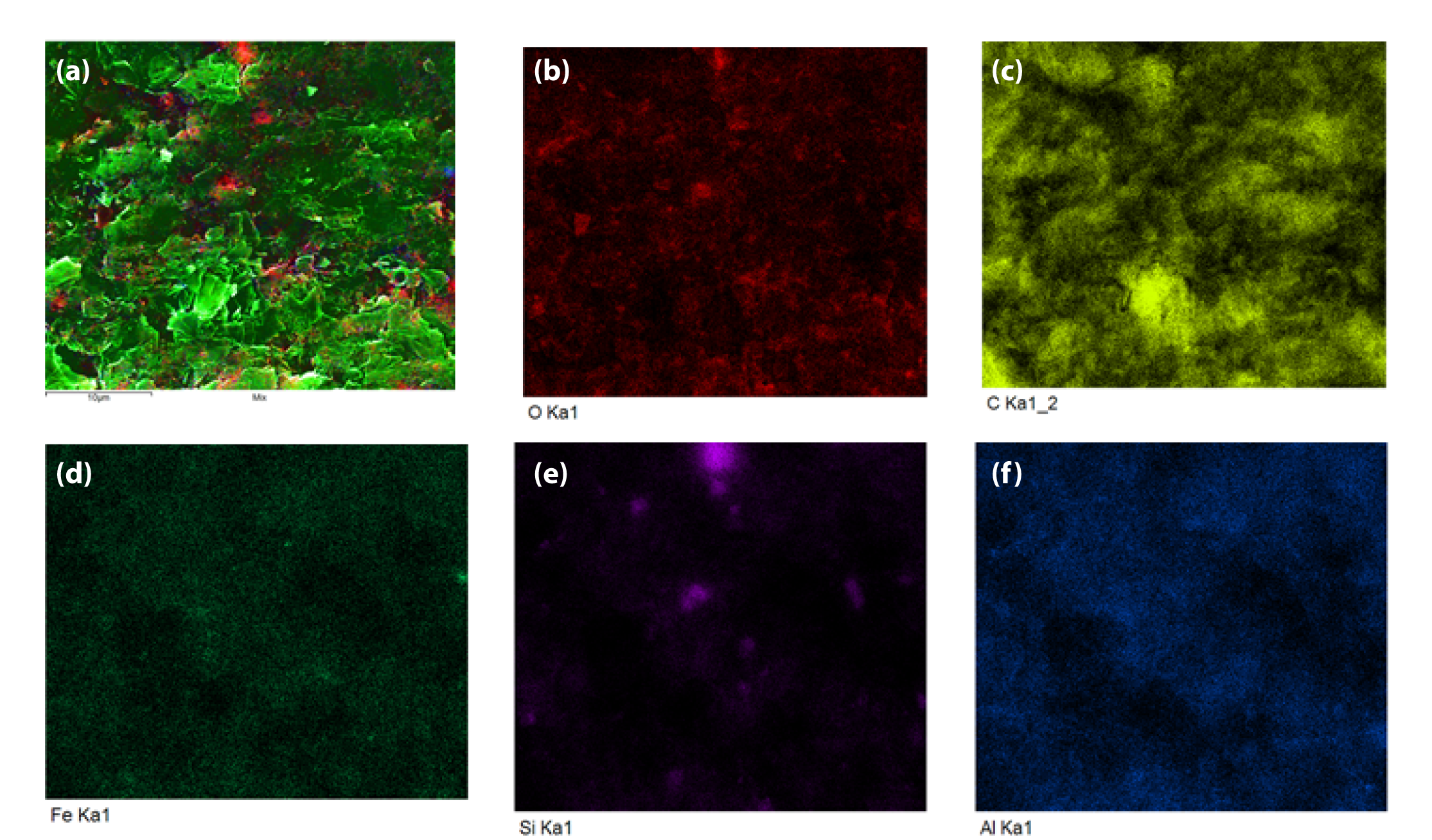

Supplement: S3 Fig — (a-f) EDX elemental mapping of PGE. (TIF) [file pone.0248142.s003.tif]

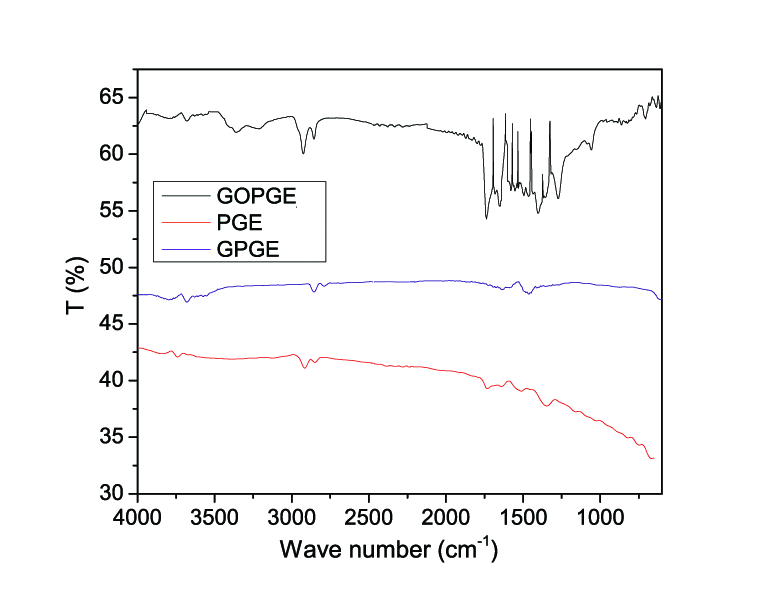

Supplement: S4 Fig — FT-IR of PGE, GOPGE and GPGE, CV was used to exfoliate PGE to GPGE, where Pt wire and Ag/AgCl were used as counter and reference electrode respectively. CV was performed at a scan rate of 50 mV s-1 from -1.0 to +1.9 V and repeated at room temperature for 20 cycles in 5.0 M HNO3. (TIF) [file pone.0248142.s004.tif]

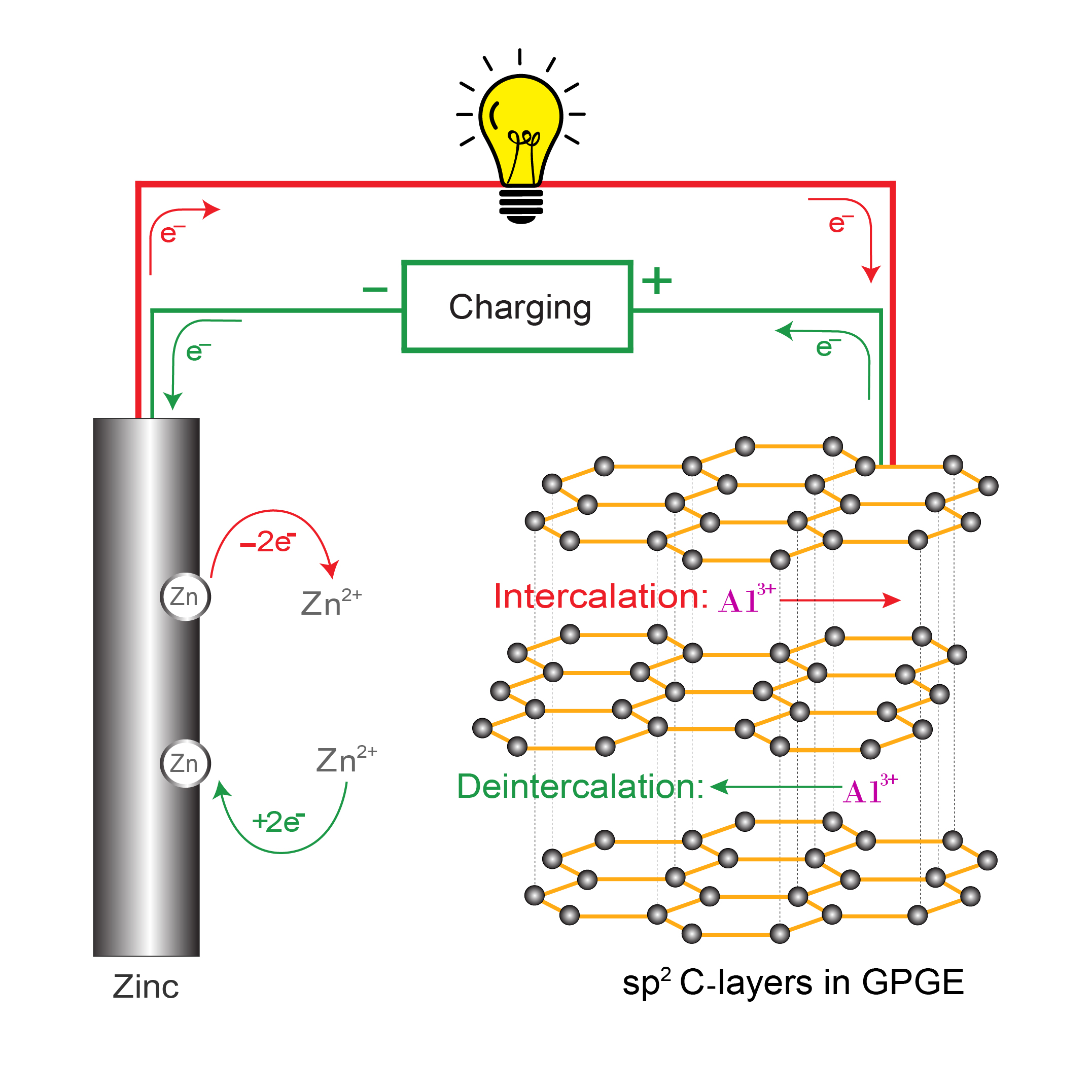

Supplement: S5 Fig — Schematic diagram of Zn/Al ion battery, where Zn is used as anode and GPGE is used as cathode and AlCl3/Zn(CH3COO)2 (0.5/0.5 M) is used as electrolytes. (TIF) [file pone.0248142.s005.tif]

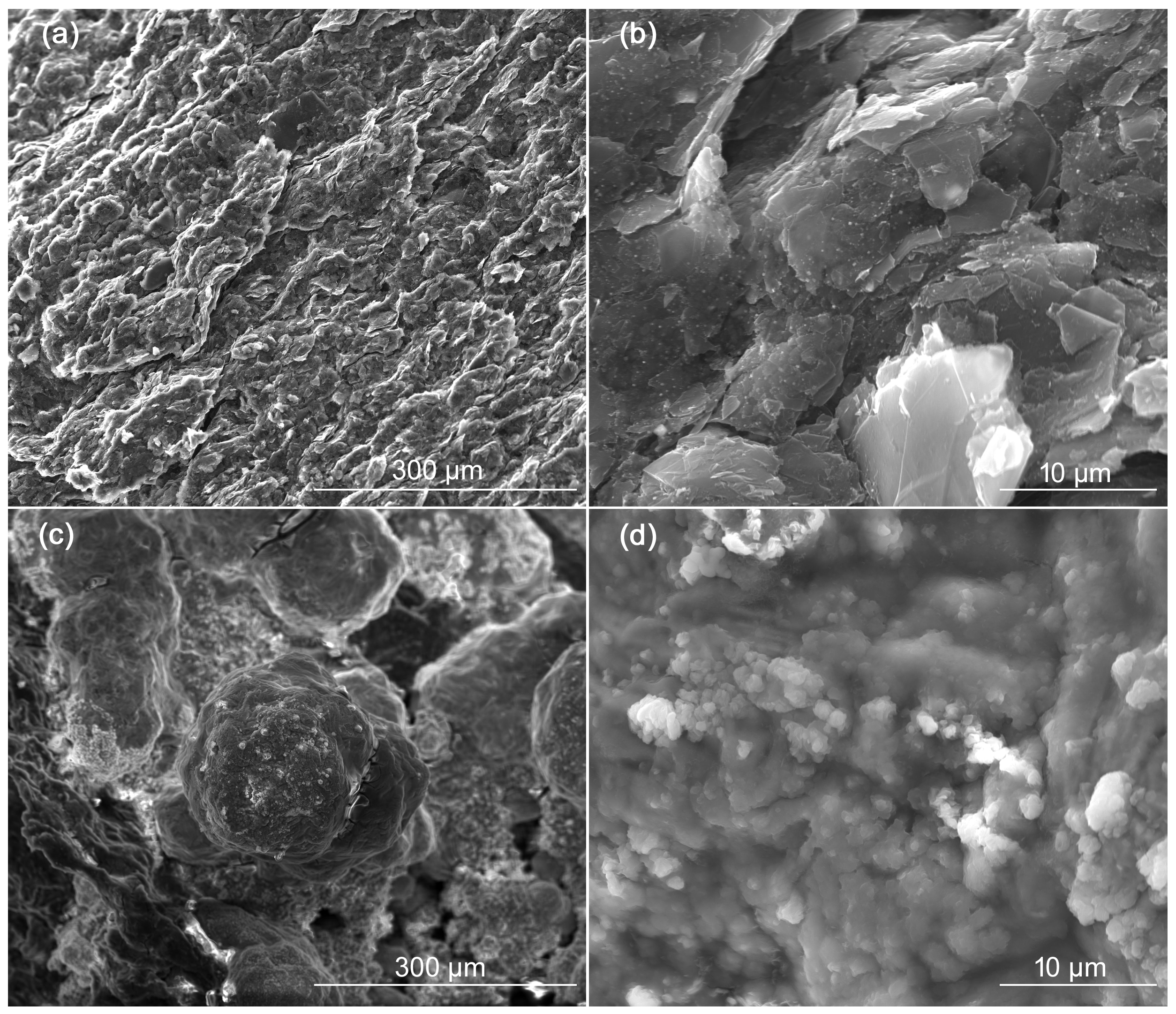

Supplement: S6 Fig — SEM images of GPGE before (a-b) and after (c-d) discharging to -0.4V (vs. Ag/AgCl). (TIF) [file pone.0248142.s006.tif]

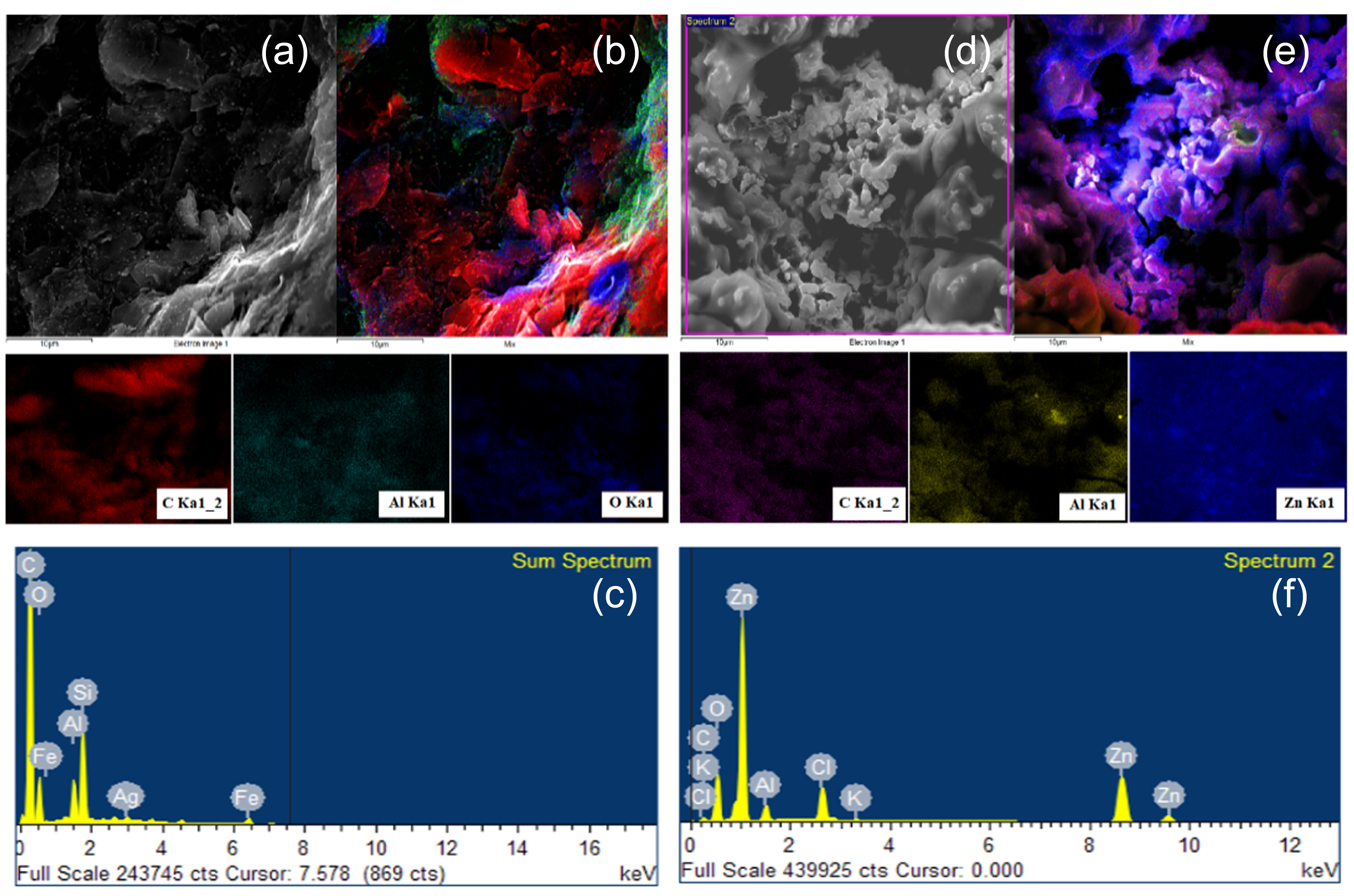

Supplement: S7 Fig — SEM and elemental mapping of GPGE before discharging (a-c) and after discharging (d-f) the cell at -0.4V (vs. Ag/AgCl). (TIF) [file pone.0248142.s007.tif]

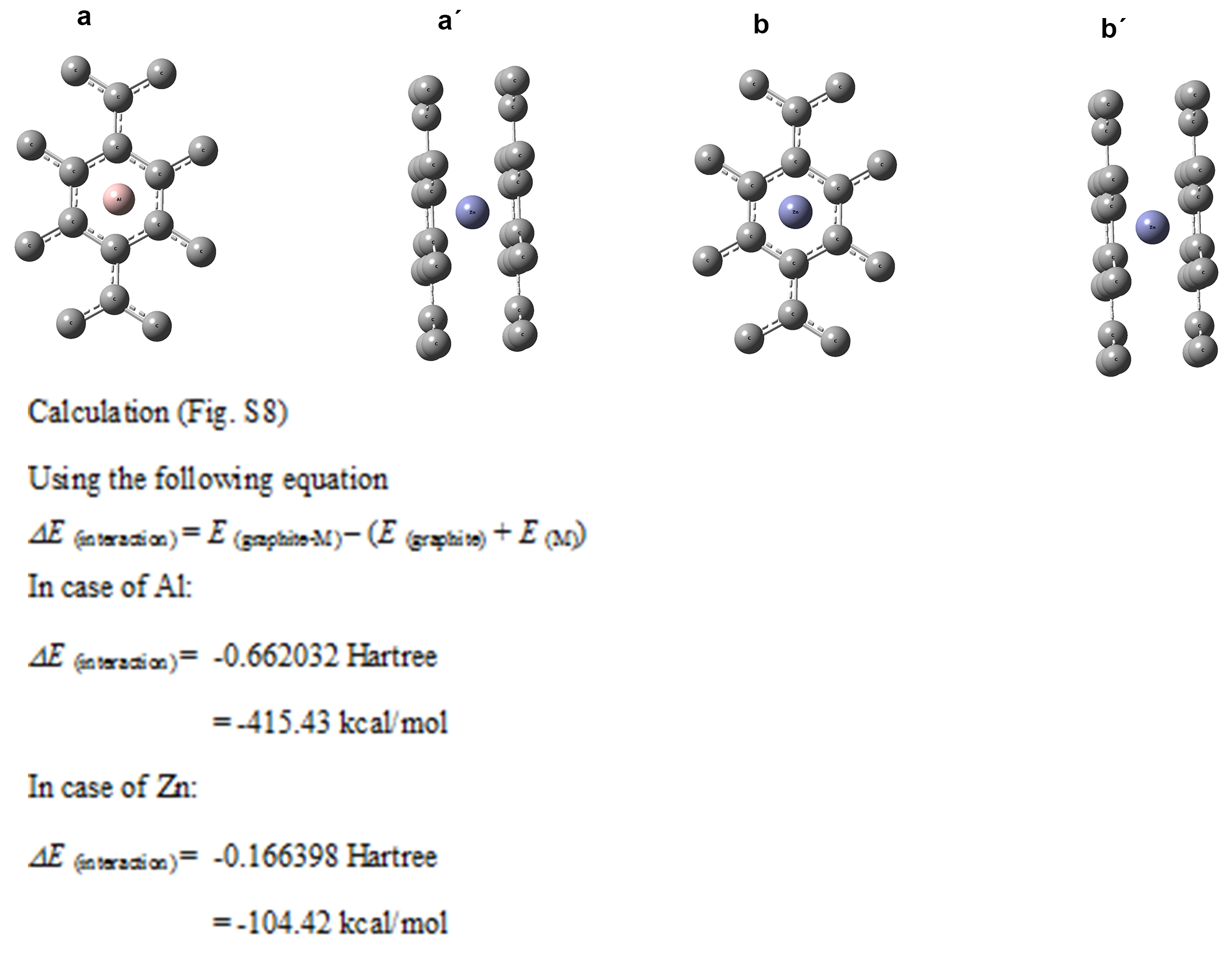

Supplement: S8 Fig — Intercalation of graphite with Al (a) top view and (a′) side view; interaction of graphite 16×16 carbon atom with Zn (b) top view and (b′) side view. (TIF) [file pone.0248142.s008.tif]

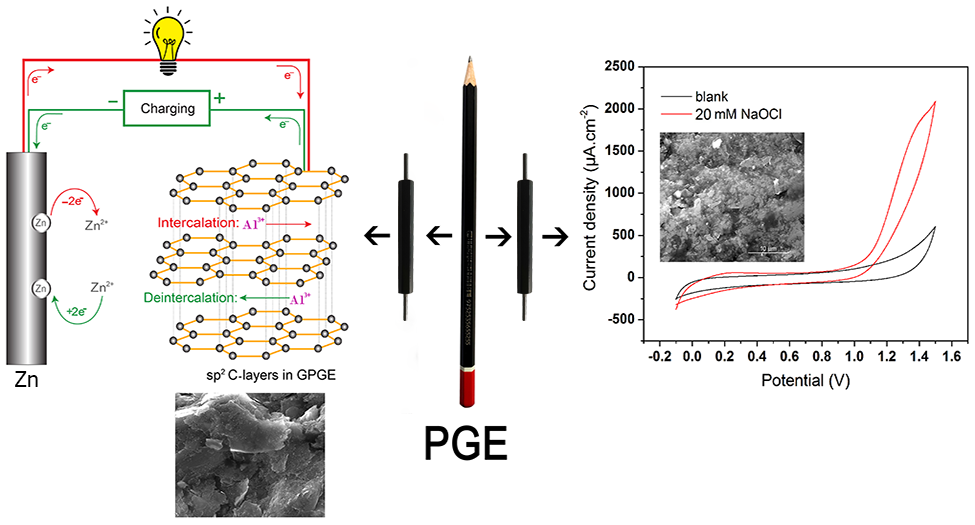

Supplement: S1 TOC — (TIF) [file pone.0248142.s011.tif]
