# Supplementary material for: Pencil graphite as electrode platform for free chlorine sensors and energy storage devices
Source: PLoS One. 2021 Mar 11;16(3):e0248142. doi: 10.1371/journal.pone.0248142 (PMC7951880; doi:10.1371/journal.pone.0248142)
Supplement: S1 Table — Comparison on the performance of different electrochemical FRC sensors. (DOCX) [file pone.0248142.s009.docx]

**S1 Table.** Comparison on the performance of different electrochemical FRC sensors.

| **Electrochemical technique** | **Electrode material** | **Linearity range**  **(mM)** | **Sensitivity**  **(µA mM^-1^ cm^-2^)** | **Response time**  **(s)** | **Stability**  **days** | **Cost / sensor**  **($)** | **Ref.** |
| --- | --- | --- | --- | --- | --- | --- | --- |
| Amperometry / Voltammetry | Au | 1.5 – 8.0 | 69.2 | 20 | 7 | 40.0 | 1 |
|  | Pt | 2.0 – 5.0 | 14.2 | 120 | 10 | 50.0 | 2 |
|  | Pyrolytic Carbon | 20 - 1410 | 170.0 | >100 | - | 5.0 | 3 |
|  | Modified PGE | 1.0 - 6.0 | 30.2 | 3 | 49 | 3.0 | 4 |
|  | PGEs | 1.0 – 8.0 | 50.0 | 3 | 14 | 0.20 | This  work |
|  | PDPE | 1.0 - 7.0 | 27.0 | 5 | 7 | 0.01 | This  work |

**REFERENCES**

1. Shekhar, H.; Chathapuram, V.; Hyun, S. H.; Seungkwan, H.; Cho, H. J. In *A disposable microsensor for continuous monitoring of free chlorine in water*, SENSORS, 2003 IEEE, 22-24 Oct. 2003; 2003; pp 67-70 Vol.1.

2. Kodera, F.; Umeda, M.; Yamada, A., Determination of free chlorine based on anodic voltammetry using platinum, gold, and glassy carbon electrodes. *Analytica Chimica Acta* **2005,** *537* (1), 293-298.

3. Meyler, R. E. P.; Edwards, M. A.; Macpherson, J. V., Exploring the suitability of different electrode materials for hypochlorite quantification at high concentration in alkaline solutions. *Electrochemistry Communications* **2018,** *86*, 21-25.

4. Pan, S.; Deen, M. J.; Ghosh, R., Low-Cost Graphite-Based Free Chlorine Sensor. *Analytical Chemistry* **2015,** *87* (21), 10734-10737.
