# Supplementary material for: Pencil graphite as electrode platform for free chlorine sensors and energy storage devices
Source: PLoS One. 2021 Mar 11;16(3):e0248142. doi: 10.1371/journal.pone.0248142 (PMC7951880; doi:10.1371/journal.pone.0248142)
Supplement: S2 Table — Comparison on efficiencies of different battery based on graphite electrodes. (DOCX) [file pone.0248142.s010.docx]

**S2 Table.** Comparison on efficiencies of different battery based on graphite electrodes.

| Sl. No. | Cathode | Anode | Electrolytes | Capacity  (mA h g^-1^) | C-rate,  (mA g^-1^) | Cyclic Stability | Discharge Voltage  (V) | Efficiency  (%) | Ref. |
| --- | --- | --- | --- | --- | --- | --- | --- | --- | --- |
| 1 | Graphite / Carbon Fiber Paper | High Purity Al-foil | AlCl_3_-Urea (1.3/1 M) | ~73.0 | 100 | ~150–200 | 1.73  (vs. Al^3+^/Al) | 99.7 | 5 |
| 2 | Pristine Graphite flakes on Tungsten-plates | Al-foil | AlCl_3_-EMIC | ≤150.0 | 100 | ~100 | 1.77  (vs. Al^3+^/Al) | 90 | 6 |
| 3 | Round Carbon paper | High purity Al-foil | AlCl_3_-EMIC (1.3/1 M) | 69.9 | 100 | ~100 | 1.8  (vs. Al^3+^/Al) | 100 | 7 |
| 4 | VO_2_ pasted on S. Steel | High purity Al-foil | AlCl_3_-Urea (1/1 M) | 116.0 | 50 | ~100 | 0.5 |  | 8 |
| 5 | Graphite on Carbon Paper | Pure Al | AlCl_3_-Urea (1.5/1 M) @120 ^ͦ^ C | ~93.0 | 100 | ~500 | 1.7 | 99 | 9 |
| 6 | Pristine Nat. graphite flakes | Al-foil  (~20 μm) | AlCl_3_-EMIC (1.3/1 M) | ~110.0 | 99 | 6000 @  60 mA g^-1^ | 2.25-2.0 & 1.9-1.5 | 98 @ 99 mA g^-1^ | 10 |
| 7 | 3D graphitic foam | Al-metal | AlCl_3_-EMIC | ~60.0 | 100 | 7500  @ 4 A g^-1^ | ~2.0 | - | 11 |
| 8 | 3D-pyrolytic graphitic foil | Al-foil | AlCl_3_-EMIC (1.3/1 M) | ~60.0 | 2000 | 4000 | ~ 1.8 | 100 | 12 |
| 9 | Natural graphite+ carbon black(10%) + PVDF(10%) on Al | Al-metal | LiPF_6_ (4M) in Ethyl methyl Carbonate | ~100.0 | 200 | 200 | 4.2 | 88 | 13 |
| 10 | Ultrathin Graphitic nanosheet | Zinc metal | Al_2_(SO4)_3_ + Zn(CH_3_COO)_2_ in H_2_O | 80.0 | 500 | ~200 | 1.0 | 94 | 14 |
| 11 | GPGE | Zn Rod | AlCl_3_ + Zn(CH_3_COO)_2_ in H_2_O | 54.0 | 55 | ~100 | ~1.1 | 95.8 | This work |

**References**

5. Angell, M.; Pan, C.-J.; Rong, Y.; Yuan, C.; Lin, M.-C.; Hwang, B.-J.; Dai, H., High Coulombic efficiency aluminum-ion battery using an AlCl_3_-urea ionic liquid analog electrolyte. *Proceedings of the National Academy of Sciences* **2017,** *114* (5), 834-839.

6. Kravchyk, K. V.; Wang, S.; Piveteau, L.; Kovalenko, M. V., Efficient Aluminum Chloride–Natural Graphite Battery. *Chemistry of Materials* **2017,** *29* (10), 4484-4492.

7. Sun, H.; Wang, W.; Yu, Z.; Yuan, Y.; Wang, S.; Jiao, S., A new aluminium-ion battery with high voltage, high safety and low cost. *Chemical Communications* **2015,** *51* (59), 11892-11895.

8. Wang, W.; Jiang, B.; Xiong, W.; Sun, H.; Lin, Z.; Hu, L.; Tu, J.; Hou, J.; Zhu, H.; Jiao, S., A new cathode material for super-valent battery based on aluminium ion intercalation and deintercalation. *Scientific Reports* **2013,** *3* (1), 3383.

9. Jiao, H.; Wang, C.; Tu, J.; Tian, D.; Jiao, S., A rechargeable Al-ion battery: Al/molten

AlCl_3_–urea/graphite. *Chemical Communications* **2017,** *53* (15), 2331-2334.

10. Wang, D.-Y.; Wei, C.-Y.; Lin, M.-C.; Pan, C.-J.; Chou, H.-L.; Chen, H.-A.; Gong, M.; Wu, Y.; Yuan, C.; Angell, M.; Hsieh, Y.-J.; Chen, Y.-H.; Wen, C.-Y.; Chen, C.-W.; Hwang, B.-J.; Chen, C.-C.; Dai, H., Advanced rechargeable aluminium ion battery with a high-quality natural graphite cathode. *Nature Communications* **2017,** *8* (1), 14283.

11. Lin, M.-C.; Gong, M.; Lu, B.; Wu, Y.; Wang, D.-Y.; Guan, M.; Angell, M.; Chen, C.; Yang, J.; Hwang, B.-J.; Dai, H., An ultrafast rechargeable aluminium-ion battery. *Nature* **2015,** *520* (7547), 324-328.

12. Zhang, X.; Tang, Y.; Zhang, F.; Lee, C.-S., A Novel Aluminum–Graphite Dual-Ion

Battery. *Advanced Energy Materials* **2016,** *6* (11), 1502588;

13. Wang, C.; Li, J.; Jiao, H.; Tu, J.; Jiao, S., The electrochemical behavior of an aluminum alloy anode for rechargeable Al-ion batteries using an AlCl_3_–urea liquid electrolyte. *RSC Advances* **2017,** *7* (51), 32288-32293.

14. Allen, M. J.; Tung, V. C.; Kaner, R. B., Honeycomb Carbon: A Review of Graphene. *Chemical Reviews* **2010,** *110* (1), 132-145.
