# Supplementary material for: Pencil graphite as electrode platform for free chlorine sensors and energy storage devices
Source: PLoS One. 2021 Mar 11;16(3):e0248142. doi: 10.1371/journal.pone.0248142 (PMC7951880; doi:10.1371/journal.pone.0248142)
Supplement: Attachment — Submitted filename: PONE comments.docx [file pone.0248142.s012.docx]

The authors have studied and reported the use of Pencil Graphite as Electrode Platform for Free Chlorine Sensors and Energy Storage Devices. While I greatly appreciate the authors for the effort taken, I feel the manuscript needs major revision to be accepted for publication.

1. Incorrect sentence formation is present at various parts of the manuscript that makes the readability of manuscript difficult. The authors need to seriously look into it.
2. The cyclic stability test was carried out only for 100 cycles and it shows a retention of 95.8%. This shows the poor efficiency of the material. The authors are recommended to carry out the test for higher number of cycles (>5000 at least).
3. In the introduction, various literatures are written (ex: Mariad, Maria et al. Aditya et al.) However, the citations to the same are not present.
4. In the materials section, it is advised to mention the name of the compounds and then write their chemical formula, instead of writing only the formula.
5. The authors claim that synthesized pencil graphite is a green material. The authors have to justify this statement.
6. The full form of PDPE to be abbreviated initially. Correct all such mistakes.
7. ‘Every day the electrode was tested for two weeks.’ No meaning and connection to the previous statement.
8. Caption of Fig. 2 appears in the middle of a paragraph, which breaks the continuity.
9. The authors could quantify FRC using UV spectroscopy and other analytical techniques for better comparison.
