# Supplementary material for: Pencil graphite as electrode platform for free chlorine sensors and energy storage devices
Source: PLoS One. 2021 Mar 11;16(3):e0248142. doi: 10.1371/journal.pone.0248142 (PMC7951880; doi:10.1371/journal.pone.0248142)
Supplement: Attachment — Submitted filename: Responses to reviewer comments.docx [file pone.0248142.s013.docx]

PONE-D-20-33482

Pencil graphite as electrode platform for free chlorine sensors and energy storage devices

The authors would like to thank the reviewers for their valuable comments and suggestions to improve the manuscript. The response to the reviewers are as follows:

Reviewer #1: This paper reports the use of pencil graphite as an electrode for dual applications that include the detection of free residual chlorine using electro-oxidation process and as an electrochemical energy storage cathode. The pencil graphite is then transferred to cellulose paper by drawing ten times and applied for the detection of free residual chlorine, which shows a sensitivity of 27 µA mM^-1^ cm^-2^ with a limit of detection of 88.9 µM and linearity up to 7
mM the work is performed systematically and it is presented nicely in the manuscript. Pencil graphite can be used for both sensor and energy-storage applications.

Responses: Thanks to the reviewer for appreciating our work.

Reviewer #2: The authors have studied and reported the use of Pencil Graphite as Electrode Platform for Free Chlorine Sensors and Energy Storage Devices. While I greatly appreciate the authors for the effort taken, I feel the manuscript needs major revision to be accepted for publication.

Responses: We would like to thank the reviewer for the critical analysis of our manuscript. Revisions have been made in the updated version of the manuscript.

1. Incorrect sentence formation is present at various parts of the manuscript that makes the readability of manuscript difficult. The authors need to seriously look into it.

Responses: English has been corrected throughout the manuscript. A copy of the manuscript with track changes has been enclosed with the submission.

2. The cyclic stability test was carried out only for 100 cycles and it shows a retention of 95.8%. This shows the poor efficiency of the material. The authors are recommended to carry out the test for higher number of cycles (>5000 at least).

Responses: In this work, we have showed Zn as anode, graphene coated PGE as cathode and a mixture of aluminum chloride (AlCl_3_) and zinc acetate (Zn(CH_3_COO)_2_) as electrolyte. In terms of safety, simplicity and cost, the proposed aqueous battery possess a huge potential as a cost effective energy storage device. However, we have only studied the cyclic stability test up to 100 cycles, which shows almost no efficiency loss. Currently, work is in progress to improve the cyclability of this battery. As these experiments are on-going, the outcome of these studies will be reported in a separate article. It is also worth mentioning that Angell et al. [1] fabricated a graphite powder based cathode material based aqueous battery with Al^3+^ as electrolyte, and reported the cyclability up to 180 cycles. Similar graphitic materials based aqueous energy storage devices have been reported by many other authors [2-6] with similar cycle life.

**References**

1. Angell, M.; Pan, C.-J.; Rong, Y.; Yuan, C.; Lin, M.-C.; Hwang, B.-J.; Dai, H., High Coulombic efficiency aluminum-ion battery using an AlCl_3_-urea ionic liquid analog electrolyte. *Proceedings of the National Academy of Sciences* **2017,** *114* (5), 834-839.

2.	Kravchyk, K. V.; Wang, S.; Piveteau, L.; Kovalenko, M. V., Efficient Aluminum Chloride–Natural Graphite Battery. *Chemistry of Materials* **2017,** *29* (10), 4484-4492.

3.	Sun, H.; Wang, W.; Yu, Z.; Yuan, Y.; Wang, S.; Jiao, S., A new aluminium-ion battery with high voltage, high safety and low cost. *Chemical Communications* **2015,** *51* (59), 11892-11895.

4. Wang, W.; Jiang, B.; Xiong, W.; Sun, H.; Lin, Z.; Hu, L.; Tu, J.; Hou, J.; Zhu, H.; Jiao, S., A new cathode material for super-valent battery based on aluminium ion intercalation and deintercalation. *Scientific Reports* **2013,** *3* (1), 3383.

5. Lin, M.-C.; Gong, M.; Lu, B.; Wu, Y.; Wang, D.-Y.; Guan, M.; Angell, M.; Chen, C.; Yang, J.; Hwang, B.-J.; Dai, H., An ultrafast rechargeable aluminium-ion battery. *Nature* **2015,** *520* (7547), 324-328.

6. Zhang, X.; Tang, Y.; Zhang, F.; Lee, C.-S., A Novel Aluminum–Graphite Dual-Ion

Battery. *Advanced Energy Materials* **2016,** *6* (11), 1502588.

3. In the introduction, various literatures are written (ex: Mariad, Maria et al. Aditya et al.) However, the citations to the same are not present.

Responses: It is fixed in the manuscript.

4. In the materials section, it is advised to mention the name of the compounds and then write their chemical formula, instead of writing only the formula.

Responses: It is fixed in the manuscript.

5. The authors claim that synthesized pencil graphite is a green material. The authors have to justify this statement.

Responses: In both the abstract and introduction section, we have claimed about the green materials as follows:

Abstract

“Therefore, pencil graphite due to its excellent electro-oxidation and conducting properties, can be successfully implemented as a low cost, disposable and green material for both sensor and energy-storage applications.”

Introduction

“So, these extremely simple, green, and low-cost pencil graphite-derived electrodes can be implemented in the future IOT sensor and energy storage applications.”

Please find below the justification of PGE as green material:

Green material involves eliminating and avoiding the use of toxic and hazardous reagents and solvents in the manufacturing and applications process [1, 2]. Pencil graphite is a biodegradable material and also recyclable. These commercial pencil graphite does not use any hazardous chemicals. In addition, using pencil drawn on to paper make the fabrication more environment friendly. In our case, we used the pencil graphite without any further modification as free residual chlorine sensor, and with a simple one-step cyclic voltammetry, it has been used as cathode in aqueous battery application. Thereby, it was termed as a green material.

Reference

## 1. [Srinivasulu Kanaparthi](https://pubs.rsc.org/en/results?searchtext=Author%3ASrinivasulu%20Kanaparthi) and [Sushmee Badhulika](https://pubs.rsc.org/en/results?searchtext=Author%3ASushmee%20Badhulika), Solvent-free fabrication of a biodegradable all-carbon paper based field effect transistor for human motion detection through strain sensing, ***Green Chem.***, 2016,**18**, 3640-3646.

## 2. https://en.wikipedia.org/wiki/Green_chemistry.

6. The full form of PDPE to be abbreviated initially. Correct all such mistakes.

Responses: These type of issues have been fixed in the manuscript.

7. ‘Every day the electrode was tested for two weeks.’ No meaning and connection to the previous statement.

Responses: Following lines are included now in the manuscript to make the connections with the statements.

“The electrode stability was tested using amperometry at an applied potential of 1.5 V vs. Ag/AgCl, with 1 mM of NaOCl added. Electrodes were tested daily over the course of two weeks; and stored at room temperature within a closed glass vial in between the measurements. Between subsequent measurements, the electrodes were cleaned in DI water and dried using nitrogen gas.”

8. Caption of Fig. 2 appears in the middle of a paragraph, which breaks the continuity.

Responses: It is fixed in the manuscript.

9. The authors could quantify FRC using UV spectroscopy and other analytical techniques for better comparison.

Responses: Currently we are working on inter laboratory and intra laboratory validation of FRC sensor, along with substrate improvement. We are hoping to publish these work in a separate paper, as currently it is in progress. As this work is the first of its kind, we have studied the behavior of PGE and PDPE as FRC sensor, as well as showed its feasibility as energy storage materials with small modification. Currently, work is in progress to improve the nature of the substrate so that these electrodes can be used in any real samples.
